# New Clinical Trials and Therapeutic Advances with Faricimab and Aflibercept 8 mg in Neovascular Age-Related Macular Degeneration: A Durability-Oriented Comparative Perspective

**DOI:** 10.3390/biomedicines14040773

**Published:** 2026-03-29

**Authors:** Feliciana Menna, Laura De Luca, Alessandro Meduri, Antonio Baldascino, Stefano Lupo, Enzo Maria Vingolo

**Affiliations:** 1 Department of Medical-Surgical Sciences and Biotechnologies, U.O.C. Ophthalmology, Sapienza University of Rome, Via Firenze 1, 04019 Terracina, Italy; stefanolupo@me.com (S.L.); enzomaria.vingolo@uniroma1.it (E.M.V.); 2 Ophthalmology Clinic, Department of Biomedical Sciences, University of Messina, 98122 Messina, Italy; laura.deluca21@gmail.com (L.D.L.); ameduri@unime.it (A.M.); 3 Ophthalmology Unit, Fondazione Policlinico Universitario A. Gemelli IRCCS, 00168 Rome, Italy; antonio.baldascino@policlinicogemelli.it

**Keywords:** neovascular age-related macular degeneration, nAMD, anti-VEGF therapy, faricimab, aflibercept 8 mg, high-dose aflibercept, angiopoietin-2, real-world evidence, retinal fluid biomarkers, treat-and-extend

## Abstract

Neovascular age-related macular degeneration (nAMD) remains a major cause of visual morbidity worldwide, although its contribution to blindness in developed healthcare systems has declined in the era of anti-VEGF therapy. Although randomized clinical trials have consistently demonstrated meaningful visual gains under structured retreatment protocols, real-world outcomes frequently decline over time due to undertreatment, limited durability, and persistent disease activity. Recent therapeutic advances have shifted the focus from maximizing short-term efficacy to engineering sustained disease control. Faricimab, a bispecific antibody targeting both VEGF-A and angiopoietin-2, introduces dual-pathway vascular modulation, while high-dose aflibercept (8 mg) enhances VEGF suppression through pharmacokinetic intensification. This perspective critically examines the biological rationale, clinical evidence, real-world implications, and strategic positioning of these agents, proposing a durability-centered framework for next-generation management of nAMD.

## 1. Introduction

Neovascular age-related macular degeneration represents the exudative and vision-threatening stage of AMD and is characterized by choroidal neovascularization, vascular leakage, and progressive macular damage [1,2,3,4,5]. The introduction of anti-VEGF therapy transformed the natural history of the disease, with landmark trials such as MARINA and ANCHOR demonstrating unprecedented visual stabilization and gain [6,7,8,9,10,11]. Subsequent development of aflibercept further optimized fixed-interval dosing strategies and broadened therapeutic flexibility [12].

However, the evolution of anti-VEGF therapy revealed a fundamental limitation: sustained disease control requires chronic and frequent intravitreal injections. In controlled clinical trials, strict dosing regimens preserve visual gains, yet real-world registry data consistently demonstrate reduced injection frequency and corresponding visual decline over time [3,4,5,6,13]. This divergence between randomized efficacy and real-world effectiveness—often referred to as the “durability gap”—has become the central therapeutic challenge in nAMD. In parallel, several alternative strategies aimed at increasing treatment durability are under investigation, including gene therapy approaches designed to enable sustained intraocular anti-VEGF expression, as well as tyrosine kinase inhibitor-based sustained delivery systems. While these approaches are beyond the primary scope of this perspective, they highlight the ongoing effort to reduce treatment burden while maintaining therapeutic efficacy.

Persistent intraretinal or subretinal fluid, incomplete anatomical response, and recurrence following interval extension contribute to long-term instability [5,14]. As a result, therapeutic innovation has shifted toward durability engineering: extending dosing intervals without sacrificing disease control. Faricimab and aflibercept 8 mg represent two distinct but complementary strategies designed to address this challenge. Faricimab is distinguished by its dual inhibition of VEGF-A and angiopoietin-2 (Ang-2), thereby targeting both angiogenic signaling and vascular instability. However, aflibercept also exhibits multi-target binding properties, as it binds VEGF-A, VEGF-B, and placental growth factor (PlGF) [10,11].

## 2. Faricimab: Dual-Pathway Vascular Modulation

Faricimab embodies a paradigm shift from single-pathway angiogenesis suppression toward vascular homeostasis modulation. While VEGF-A remains the dominant driver of choroidal neovascularization in nAMD, the angiopoietin–Tie2 signaling axis has emerged as a critical regulator of endothelial stability, vascular permeability, and inflammatory amplification [7,12].

Under physiological conditions, Angiopoietin-1 activates the Tie2 receptor, promoting endothelial quiescence, tight junction integrity, and vascular stabilization. In contrast, Angiopoietin-2 (Ang-2) acts as a context-dependent antagonist of Tie2 signaling. Under hypoxic and inflammatory conditions—hallmarks of neovascular AMD—Ang-2 is upregulated, leading to endothelial destabilization, increased permeability, leukocyte adhesion, and amplification of VEGF-driven angiogenesis [15]. Elevated intraocular Ang-2 levels have been associated with persistent vascular leakage and disease activity in retinal vascular disorders [14,16].

Faricimab simultaneously neutralizes VEGF-A and Ang-2, aiming not only to suppress angiogenesis but also to restore vascular stability through rebalancing Tie2 signaling [7,17]. This dual inhibition model proposes that VEGF suppression alone may be insufficient in cases where vascular instability and inflammatory signaling contribute to recurrence. Preclinical models demonstrated that combined VEGF-A and Ang-2 inhibition resulted in greater reduction in leakage, improved vascular normalization, and enhanced endothelial barrier function compared to VEGF blockade alone [16].

Although the biological rationale for Ang-2 inhibition is compelling, its translation into clinically meaningful additive benefit has not been consistent across studies. In this regard, the Phase 2 RUBY trial did not demonstrate a significant advantage of Ang-2 pathway inhibition, highlighting the complexity of translating preclinical findings into clinical outcomes [15]. Accordingly, the extended durability observed in the TENAYA and LUCERNE trials may reflect not only dual-pathway inhibition but also differences in molar dose and ocular pharmacokinetics compared with aflibercept 2 mg [10]. Faricimab, administered following loading doses with personalized treatment intervals of up to 16 weeks, achieved non-inferior visual acuity gains compared with aflibercept 2 mg administered every 8 weeks. Aflibercept 2 mg represents the standard approved intravitreal dosing regimen, typically given every 8 weeks after an initial loading phase. Approximately 44–45% of patients were maintained on 16-week dosing intervals at year one, and two-year follow-up confirmed sustained visual and anatomical outcomes, with a substantial proportion of patients remaining on extended dosing regimens [17]. Notably, these durability benefits were achieved with comparable reductions in central retinal thickness and fluid resolution.

These findings suggest that dual-pathway modulation may extend disease quiescence beyond traditional VEGF suppression thresholds. Durability modeling indicates that stabilization of endothelial junctions via Tie2 signaling may reduce recurrence risk even as VEGF levels fluctuate near therapeutic thresholds. In this framework, faricimab’s advantage may derive not solely from longer pharmacokinetic presence, but from modulation of vascular behavior [18].

Real-world data are beginning to explore this hypothesis. Observational switching studies in treatment-experienced eyes with persistent fluid despite frequent anti-VEGF therapy have reported reductions in intraretinal fluid, extension of injection intervals, and stabilization of visual acuity following transition to faricimab [19,20,21]. In several cohorts, patients previously requiring 4–6-week intervals achieved meaningful extension beyond 8 weeks. While these studies are limited by retrospective design and short follow-up, they provide preliminary evidence that dual inhibition may benefit refractory phenotypes.

Registry-based early analyses suggest that faricimab maintains durability outside protocol-driven environments, although confirmation through large multicenter datasets is still pending [19]. Importantly, whether Ang-2 inhibition influences long-term structural remodeling—such as fibrosis formation or progression of atrophy—remains an open and clinically significant question. Chronic endothelial instability has been implicated in fibrotic scar development; thus, theoretical benefits of vascular stabilization extend beyond fluid control. Longitudinal imaging analyses will be required to determine whether dual-pathway modulation alters the trajectory of structural degeneration.

Another relevant consideration is heterogeneity of disease biology. Not all patients exhibit identical patterns of angiogenic drive or inflammatory activation. Some eyes may be predominantly VEGF-driven, whereas others may demonstrate a greater contribution from vascular destabilization and inflammatory signaling. Faricimab’s biological diversification may offer particular benefit in the latter subgroup. However, validated biomarkers to stratify patients according to Ang-2-dominant phenotypes are not yet available. Integration of imaging biomarkers—such as OCT-derived intraretinal and subretinal fluid, hyperreflective foci, and choroidal thickness—and molecular profiling, including VEGF and Ang-2 expression levels or inflammatory cytokine signatures, may eventually clarify patient selection.

Safety data from TENAYA and LUCERNE did not reveal increased rates of intraocular inflammation or systemic adverse events compared with aflibercept [10]. Given that both faricimab and high-dose aflibercept are administered at relatively high molar doses, careful consideration of intraocular inflammation is warranted. Available clinical trial data suggest a generally favorable inflammatory safety profile for both agents, although continued post-marketing surveillance and real-world data will be essential to fully characterize their long-term safety.

Strategically, faricimab represents a mechanistic expansion rather than a pharmacokinetic intensification. It introduces pathway diversification into retinal therapy, potentially broadening the biological scope of disease control. Whether this translates into superior long-term remodeling or merely equivalent durability achieved through different means remains to be determined through comparative effectiveness research.

## 3. Aflibercept 8 mg: Pharmacokinetic Intensification

High-dose aflibercept (8 mg) represents a conceptually distinct strategy in the evolution of anti-VEGF therapy. Rather than diversifying molecular targets, it intensifies VEGF pathway suppression by increasing intravitreal molar exposure fourfold relative to the standard 2 mg formulation [9,22]. Aflibercept binds three ligands—VEGF-A, VEGF-B, and PlGF—although the relative clinical relevance of VEGF-B and PlGF inhibition in neovascular AMD remains uncertain [12]. Its established efficacy and safety profile over more than a decade of clinical use provided a strong foundation for dose escalation.

The rationale for 8 mg dosing arises from pharmacokinetic and pharmacodynamic modeling demonstrating that durability is closely linked to the duration for which intraocular VEGF levels remain suppressed below a critical threshold [22]. With standard 2 mg aflibercept, VEGF suppression gradually diminishes as intravitreal concentration declines, leading to recurrence of exudation once therapeutic thresholds are exceeded. Increasing the injected molar dose extends the period during which intraocular drug levels remain above this threshold, thereby prolonging effective VEGF blockade without altering receptor-binding characteristics.

This strategy reflects a shift from mechanistic innovation toward pharmacologic optimization. It presumes that VEGF remains the principal driver of neovascular activity in most patients and that insufficient duration of suppression—rather than incomplete pathway coverage—accounts for recurrence in a substantial proportion of cases.

The Phase III PULSAR trial provided the pivotal evidence supporting this approach [9]. In this randomized study, aflibercept 8 mg administered every 12 or 16 weeks was compared with aflibercept 2 mg every 8 weeks following loading doses. At one year, visual acuity outcomes were non-inferior across groups, with mean gains comparable to historical anti-VEGF benchmarks, meaning that visual acuity and anatomical results were comparable between the treatment groups. Importantly, a significant proportion of patients maintained 12- or 16-week dosing intervals without evidence of loss of anatomical control. Central retinal thickness reductions and fluid resolution were similar between high-dose and standard-dose arms, supporting the hypothesis that intensified VEGF suppression can extend durability without compromising efficacy [9,19].

From a biological perspective, intensified VEGF blockade may provide more consistent suppression of neovascular endothelial proliferation and vascular permeability. Unlike dual-pathway modulation, this approach does not directly target vascular instability mediated by Ang-2 or inflammatory signaling. Instead, it reinforces the centrality of VEGF-driven angiogenesis in disease maintenance. Whether this suffices in eyes with chronic inflammatory or fibrotic components remains to be clarified.

One theoretical advantage of the 8 mg strategy is mechanistic continuity. Clinicians familiar with aflibercept 2 mg retain a well-established safety and efficacy framework, reducing uncertainty associated with introducing novel pathway modulation. Additionally, pharmacokinetic intensification avoids potential complexity of pathway cross-regulation and maintains a predictable biological profile.

However, durability achieved in randomized trials must translate into routine clinical practice to meaningfully alter outcomes. Early real-world experience with aflibercept 8 mg, though limited by short follow-up, suggests that patients previously requiring frequent 2 mg injections may achieve interval extension after switching to the higher dose [23]. Observational cohorts report stable anatomical control and maintenance of vision with extended intervals, particularly in eyes demonstrating recurrence under 2 mg regimens. Whether these interval extensions are sustained beyond one year in heterogeneous real-world populations remains an essential research question.

Another important consideration is heterogeneity of response. Registry analyses indicate that approximately one-third of patients require sustained high-frequency injections even under treat-and-extend protocols [5]. It remains unclear whether this subgroup reflects intrinsic biological resistance to VEGF suppression or suboptimal duration of blockade. High-dose aflibercept may benefit patients whose recurrence is primarily threshold-dependent rather than pathway-diversification dependent. Comparative effectiveness studies between 8 mg aflibercept and faricimab in refractory populations will be particularly informative.

Safety remains a central concern in any dose-escalation strategy. The clinical experience with brolucizumab further illustrates that highly potent and sustained VEGF-A inhibition can achieve substantial durability. However, its use has been limited by intraocular inflammatory complications, emphasizing the importance of balancing efficacy with safety in the development of long-acting therapies [24]. The PULSAR trial did not demonstrate increased rates of intraocular inflammation, retinal vasculitis, or systemic thromboembolic events compared to standard dosing [9]. Nevertheless, broader post-marketing surveillance in elderly populations with cardiovascular comorbidities will be necessary to confirm systemic safety across larger datasets [25]. Because systemic VEGF suppression has theoretical associations with vascular events, pharmacovigilance will be critical as cumulative exposure increases.

Beyond durability, the long-term structural consequences of intensified VEGF suppression warrant investigation. Chronic VEGF inhibition has been hypothesized to contribute to macular atrophy progression in some contexts, although causal relationships remain debated [26]. Whether prolonged high-level suppression with 8 mg dosing influences atrophic remodeling differently from standard regimens requires longitudinal imaging analysis.

Strategically, aflibercept 8 mg positions itself as an evolutionary rather than revolutionary innovation. It reinforces the VEGF axis as the dominant therapeutic target while addressing the principal limitation of prior regimens: insufficient duration. In doing so, it offers clinicians a familiar mechanism combined with extended dosing flexibility. 

These findings highlight that faricimab and aflibercept 8 mg represent two complementary but distinct durability strategies (Table 1).

## 4. Real-World Durability and Heterogeneity of Response

The discrepancy between randomized clinical trial outcomes and real-world effectiveness remains a major challenge in nAMD management. While pivotal studies report sustained visual gains under protocol-driven regimens [2,8,9], large observational datasets consistently show lower injection frequency and progressive attenuation of visual outcomes over time [3,4,5,6,13]. Registry analyses from the Fight Retinal Blindness! database, the UK EMR Users Group, and other multicenter cohorts demonstrate that patients frequently receive fewer injections than recommended, with corresponding reductions in long-term visual maintenance [4,5,6]. These findings highlight that treatment sustainability in clinical practice is a key determinant of functional outcomes.

Undertreatment is multifactorial, driven by clinic capacity constraints, patient comorbidities, injection fatigue, caregiver burden, and healthcare system limitations [3,4]. In this context, the ability to maintain extended dosing intervals without compromising anatomical control is central to real-world effectiveness.

Early real-world data on faricimab suggest potential benefits in treatment-experienced populations. Switching studies in patients with persistent intraretinal or subretinal fluid despite regular anti-VEGF therapy have reported anatomical improvement and extension of injection intervals following transition to faricimab [19,20,21]. Reductions in central retinal thickness and improved fluid control have been observed in cohorts previously requiring frequent dosing. Although limited by retrospective design and short follow-up, these findings suggest that dual-pathway inhibition may benefit refractory phenotypes. The contribution of Ang-2 blockade to vascular stabilization beyond VEGF suppression remains biologically plausible but requires confirmation in larger longitudinal datasets.

Real-world evidence for aflibercept 8 mg is still emerging. Preliminary reports indicate that patients previously requiring frequent aflibercept 2 mg injections may maintain longer intervals after switching to 8 mg therapy [25]. This pharmacokinetic intensification may partially address undertreatment patterns observed in registry studies [4,13]. However, comparative real-world data between high-dose aflibercept and faricimab are currently lacking, and head-to-head analyses will be necessary for optimal therapeutic positioning.

Beyond injection frequency, heterogeneity of treatment response remains a key issue. Persistent intraretinal fluid is consistently associated with worse long-term visual outcomes [14,26], whereas limited subretinal fluid may not uniformly predict functional decline. These differences likely reflect underlying variability in angiogenic drive, inflammatory signaling, and vascular remodeling. Real-world data indicate that a substantial proportion of patients require sustained high-frequency treatment despite treat-and-extend strategies [5], raising the question of whether these individuals may benefit more from pathway diversification or intensified VEGF suppression.

Structural remodeling over time represents an additional unmet need. Fibrosis and progressive atrophy may occur despite fluid control, suggesting that current approaches do not fully preserve retinal integrity [27]. Longitudinal imaging studies will be required to determine whether dual-pathway inhibition modifies these processes differently from intensified VEGF blockade.

Finally, safety monitoring in real-world populations remains essential. Clinical trials have reported no increase in systemic adverse events with these agents [9,25]. However, broader populations include older patients with significant comorbidities. Ongoing pharmacovigilance and registry-based analyses will be critical to confirm long-term safety.

## 5. Translational Implications and Unmet Needs

Reducing treatment burden is not merely a logistical objective but a key determinant of long-term visual outcomes. Undertreatment in real-world practice is consistently associated with visual decline [4,6], underscoring the importance of therapeutic strategies capable of maintaining extended dosing intervals. Agents that sustain 12–16-week intervals may therefore have meaningful implications at the population level.

However, extended dosing alone does not address all unmet needs. Geographic atrophy may progress independently of VEGF suppression [25], and a subset of patients continues to exhibit incomplete response despite intensified therapy [26]. In addition, predictive biomarkers for long-term structural remodeling remain insufficiently developed.

Emerging therapeutic approaches include sustained-release delivery systems [16], complement inhibition strategies [28], and the integration of artificial intelligence-guided adaptive dosing models [29] and gene therapy targeting intraocular VEGF expression [30]. Future treatment paradigms will likely combine long-acting pharmacologic agents with precision-driven monitoring strategies.

While current therapies primarily target exudation control, long-term structural outcomes such as macular atrophy and fibrosis remain critical challenges. Addressing these processes may require more selective pathway modulation, including strategies involving pigment epithelium-derived factor (PEDF) or complement pathways, which have shown potential in modulating retinal degeneration and inflammation [31,32].

## 6. Conclusions

In conclusion, both faricimab and aflibercept 8 mg represent significant advances in durability-oriented therapy for neovascular AMD. However, the relative contributions of target specificity, molar dosing, and pharmacokinetics remain to be fully elucidated. Future research should focus on long-term safety, optimal patient selection, and the impact of these therapies on structural retinal outcomes beyond fluid control.

## Figures and Tables

**Table 1:** Analytical Comparative Framework: Faricimab vs. Aflibercept 8 mg in nAMD.

Dimension	Faricimab	Aflibercept 8 mg	Clinical Interpretation
Therapeutic Paradigm	Pathway diversification	Dose intensification	Two distinct durability strategies
Primary Biological Axis	VEGF-A + Ang-2 (Tie2 modulation)	VEGF-A (high molar suppression)	Diversification vs. amplification
Endothelial Stability	Direct modulation via Ang-2 blockade	Indirect via prolonged VEGF suppression	May matter in refractory phenotypes
Inflammatory Crosstalk	Potential reduction via Ang-2 inhibition	No additional inflammatory pathway modulation	Possible relevance in chronic fluid
Pharmacokinetic Strategy	Standard molar exposure	4× molar concentration vs. 2 mg	Longer time above VEGF threshold
Durability Modeling Concept	Reduced recurrence propensity via vascular normalization	Extended VEGF suppression duration	Stability via different biological logic
Phase III Trials	TENAYA/LUCERNE	PULSAR	Both non-inferior to aflibercept 2 mg
Max Evaluated Interval	16 weeks	16 weeks	Comparable RCT durability ceiling
Year 1 ≥ 16-week Interval	~45–50%	Substantial proportion ≥ 12–16 weeks	Similar durability signal
Switching Data (RWE)	Fluid reduction in persistent IRF cases [19,26]	Extension after frequent 2 mg dosing [20]	Different real-world niches emerging
Phenotype Hypothesis	Vascular instability/persistent IRF	VEGF-threshold recurrence	Requires biomarker validation
Structural Remodeling Hypothesis	Possible impact on fibrosis via vascular stabilization	Unknown long-term atrophy impact	Longitudinal imaging needed
Systemic Exposure Concern	Standard anti-VEGF systemic profile	Higher cumulative VEGF blockade	Long-term surveillance essential
Strategic Positioning	Mechanistic expansion	Evolutionary optimization	Complementary, not mutually exclusive

## Data Availability

No new data were created or analyzed in this study. Data sharing is not applicable to this article.

## Abbreviations

The following abbreviations are used in this manuscript:

AMD	Age-Related Macular Degeneration
Ang-2	Angiopoietin-2
APC	Article Processing Charge
CNV	Choroidal Neovascularization
IRF	Intraretinal Fluid
nAMD	Neovascular Age-Related Macular Degeneration
OCT	Optical Coherence Tomography
PlGF	Placental Growth Factor
RCT	Randomized Controlled Trial
RWE	Real-World Evidence
T&E	Treat-and-Extend
VEGF	Vascular Endothelial Growth Factor
VEGF-A	Vascular Endothelial Growth Factor A
